# Management of traumatic injury prevention in sports training and competition

**Published:** 2012

**Authors:** DC Diaconescu, D Ferechide

**Affiliations:** * Faculty of Sciences, Medical Assistance Specialty, University of Piteşti, Argeş; ** “Carol Davila” University of Medicine and Pharmacy, Bucharest

**Keywords:** morphometric measurements, sports injuries

## Abstract

The paper had as an applicative objective the epidemiological analysis of the appearance of traumatic injuries in athletes during competitions and trainings.

In the period the athletes have been followed (one year), 426 traumatic injuries have been registered in 87 athletes who were analyzed in the study. Most of them were mild injuries, to which recovery has been made quite rapidly.

The knee was the most affected of the joints, followed by the joints of the fingers from the hand, the ankle and hip.

Moreover, the distribution of the cases of traumatic injuries was followed, as it was done according to the way they happened: by direct traumatism (collision with another player, with the ball or the ground due to a fall) or by mechanic overload with indirect injuries (stress injuries, including muscular eccentric/ plyometric contractions).

Most of the traumatic injuries are recurrent, this fact being explained by the raise in the vulnerability of the affected segment when exposed to traumatic agents, probably because of some insufficient processes of recovery or/ and the early resumption of physical activity.

The prevention of traumatic injuries in sports is based on the following:

➢ a good physical preparation and an optimum adaptation of the organism to the specific physical effort in this sports field;
➢ avoiding excessive training (of the overtraining)
➢ an environment which assures the security of the athletes;
➢ an optimum recovery of the previous injuries;
➢ a specialized observation of the sports activity;
➢ strengthening the security rules and mastering the adequate technique;
➢ quality medical-sports selection and the biological preparation for the contest;
➢ quality management of the sports activity.

## Introduction

Getting sports performances cannot be obtained only by training; it also includes adequate eating habits, medication, physical and psychological recovery, the prevention and recovery of the injuries [**1**]. Consequently, the amount of people involved in the process of obtaining performance raises, we are taking about an interdisciplinary team that includes the trainer, physiotherapist, nutritionist, psychologist, biochemist, sports medicine physician [**3**].

Maintaining the state of health of the athletes and, as a result, the increase of the performances, is possible only by a tight relationship between all the interested factors, the main objective being not only the treatment of some traumatic disorders or of hyperfunctional nature mainly in the reversible state, but also the prevention of their production by elaborating an optimized strategy by the professional team [**4**].

**The sports environment is certainly the place diverse types of traumatisms are most often met [9]. According to their severity, they can temporarily hinder or sometimes definitively put an end to a sports career. That is why, these traumatisms are less important for amateur athletes, but have a major impact on the performance athletes [11].**

Based on the diagnosis of the physical development, a series of very important recommendations both in the initial selection and at the periodic checkups of all the athletes can be done.

**Theory**

**A. The classification of the traumatisms in sports**

The traumatisms in sports are classified in three types according to the specialty literature: *macrotrauma injuries, microtrauma injuries and hyperfunctional injuries [**8**].*

**We should also mention the visceral type conditions: the most frequently met are the pains in the hepatobiliary area (mostly in women) [20].**

Another classification of the traumatisms is done according to the anatomical structure, which splits them into two distinct forms: *traumatisms of the hard parts (bone structures) *and *traumatisms of the soft parts *(muscular and joint injuries) [**22**].

According to the risk of accident by collision, the sports are classified in three categories in the specialty literature:

1. *Contact/ collision sports: *basketball, football, handball, boxing, water jumps, ice hockey, judo, skiing (jumps), martial arts, polo, horse riding, etc.

2. *Limited contact sports: *cycling, athletics, gymnastics, volleyball, weightlifting, freestyle skiing, alpine skiing (descent, slalom), etc.

3. *Sports without contact: *shooting, badminton, bodybuilding, ballroom dance, golf, curling, touristic orientation, etc. [**10,17**].

**B. The risk factors involved in the traumatic accidents occurrence in athletes, according to the practiced sport**

**- The play field influences the risk of accidents occurrence due to the improper installation of the devices.**

**- The unfavorable weather conditions can directly influence the security state of the athlete [3].**

**- Inappropriate equipment/gear [21]**

**- Unsportsmanlike behavior of the competition [24]**

**- Inappropriate nutrition [5,6]**

**- Medication [15,20]**

**- Incorrect technique [8,12]**

**- Frequent training [15,16]**

**- Incorrect warm-up [2,7]**

**- Lack of focus [23]**

**- Indiscipline [7]**

**- Incorrect organization of the medical check-up [20]**

**- Prophylactic management in sports traumatology [13,14]**

C. **The prophylaxis of the traumatisms can be classified as primary, secondary and tertiary.**

The examples of ***primary prophylaxis ***include the prophylactic dressing (tapping and strapping) no matter the anterior traumatisms. ***The secondary prophylaxis ***is represented by the precocious diagnosis of a traumatism and the qualified intervention of preventing the installation of a disability and the reduction of the incidence of traumatism reappearance, while the ***tertiary prophylaxis ***is focused on the recovery in order to correct the existent disabilities, which are considered favoring factors in the appearance of a traumatism [**18,20**].

D. **The means for recovery** can be classified as it follows:

1. According to the effects

- Neuropsychological;

- Neuromuscular;

- Endocrine-metabolic;

- Cardio respiratory.

2. According to the membership of the means of recovery

- Balneo physiotherapy (warm hydrotherapy, sauna, massage – auto-massage, natural-artificial oxygenation, natural – artificial negative air ionization, acupuncture-acupressure, yoga, etc.);

- Psychotherapy (suggestion-autosuggestion, autogenic training, neuromuscular relaxation, neurotropic-psychotropic medication);

- Diet products (alkalizing, fluid sugar, rich in vitamins and microelements, caloric norm, etc.);

- Pharmacological (compensatory, substitutive);

- Active resting-passive resting (sleep).

**PERSONAL CONTRIBUTION**

**Purpose of research**

The necessity of doing a statistical analysis of the traumatology aspects that are found in different sports fields, respectively of the groups we want to study, represents the main reason for choosing this subject.

**General objectives**

- establishing the incidence of traumatisms in different sports fields;

- detecting and reducing the internal and external favouring factors;

- establishing a program of prophilactic exercices;

- establishing some methods through which we can decrease the amount of serious traumatisms according to their production mechanism;

- subsequent follow-up for a period of time (2 years) of the incidence of traumatisms and the elaboration of conclusions.

**Research hypotheses**

The study starts from the idea that the high incidence of the traumatisms in the studied sports fields has appeared due to some associations of internal and external factors, which can be counteracted.

The external factors are the following: the improper state of the field, methodical mistakes in the training process, and the brutality of the opponents.

The internal factors are the following: anterior traumatisms which were uncompletely recovered, hypocalcemias, stress bone, microtraumatisms, etc.

**Stages of the research**

*The study of the present speciality bibliography *mainly contains the methods of analysis of the distribution of the locomotor apparatus in relation to diverse risk factors.

*The achievement of the experimental methodology, *choosing a representative sample (the experimental group); establishing the working variables: the variable dependant on bone, joint and muscle traumatic injuries, with different localizations at the level of the body segments), according to the classificatory independent variables (age, size, weight, triggering factors, circumstances of appearance, period of training and competition, existence of relapse, etc.).

*The experiment *and the follow-up period are specific for the modification of the dependant variable (the frequency of appearance of the main types of muscular injuries) in the selected group, in a certain period of time (1 year).

*The analysis and discussion of the results obtained: *determining the correlations between the frequency of different types of traumatic lesions of the locomotor apparatus and the independent classificatory variables mentioned.

Drawing conclusions and proposals.

## Material and Method

**Bibliographical method.** The bibliographical documentation represents the study of the existent knowledge in the worldwide specialty literature. In the case of the proposed study, we have appealed to the scientific documentation based on some sources in the following fields: physical education and sports, psychology, pedagogy, sports medicine, sociology, etc.

**Observation method.** In the process of the athletes’ evolution follow-up in trainings and competitions, starting from the need of knowing the sports environment in which they perform their activity, we have tried to highlight the potential risks of appearance of some accidents, favored by certain environment complexes.

**The tests/ experimental method** targets the accurate determination of the physical or psychical assimilation level of development. The determination of the main morphometric indicators such as the weight, waist, body mass index, represents the time required in any experimental approach. The importance of these parameters resides in the fact that they allow the ranking of the subjects, a follow-up in the dynamics of their evolution from the physical point of view, an overall assessment of the growth and development level, of the nutritional status, etc.

**The statistical-mathematical method.**

The statistical-mathematical processing of the data has respected the methodological exigencies that were anteriorly exposed and was done by using Microsoft Excel. Moreover, the graphs were edited with the above-mentioned program, according to the type of variables manipulated during the experiment.

**Morphometric parameters**

1. Height

2. Bust

3. Length of the arms

4. Length of the legs

5. Biacromial diameter

6. Transverse thoracic diameter

7. Bitrochanteric diameter

8. Scale

9. Thorax area

10. Abdominal area

11. Arm area

12. Forearm area

13. Thigh area

14. Leg area

15. Antroposterior thoracic diameter

16. Determination of the adipose tissue covers

**Testing the ability of doing aerobic exercises**

The functional parameters of the respiratory and cardiovascular apparatus are studied, the most conclusive parameter for the testing of the ability of doing aerobic exercises, is unanimously considered the maximum amount of oxygen consumption (VO2 max).

There are two categories of tests used to determine the ability of doing aerobic exercises.

A. Cardiovascular functional explorations

These laboratory tests investigate the evolution of the cardiac frequency (CF) and of the blood pressure (BP) at rest, standardized effort, and return. The tests that are more often used are the following: Schellong test, Martinet test and Ruffier test.

B. Determining the ability of doing effort in the laboratory

It is done by direct methods (spiroergometry test) or indirect (Astrand test).

**Testing the ability of doing aerobic exercises**

It is realized in the laboratory through indirect methods, which highlight the efficiency of the muscular activity in conditions of anaerobiosis.

The most often used tests are the following: Miron Georgescu test, Sargent test, Wingate test, Bosco test and TLR test (the total labor realized).

**Testing the capacity of effort on the field**

Allows obtaining the most real image of the possibilities of the athlete who is explored in his familiar environment. The functional tests include the clino-orthostatic test (Schellong); Ruffier test; calculating the Dorgo recovery index, etc.

**Research development (experimental)**

The research took place during 2010 and 2012 on a lot of performance athletes in three different teams of sports (handball, volleyball, football), with certain age and sex particularities, who activate in an upper echelon. The realizing of a lot of 142 athletes was necessary for the experimental group.

The difficulties the couches and the athletes are dealing with in the training process, from the point of view of the risks of accidents, have been included in the study.

**Material**

The structure of the sample lot shows a medium age of 21,5 years, being relatively homogeneous from an anthropometric and functional point of view (Coefficient Variability = 13,7%), without significant personal pathological antecedents or current health problems.

**Method**

The athletes have been followed and investigated periodically and thoroughly by an anamnesis interview, in which they were asked to talk about the appearance of possible accidents, the circumstances of those accidents, the period of temporary retirement from the sports activity, the treatment followed (including the one of recovery) and the posttraumatic evolution. We have closely followed the trainings in which these athletes have taken part, as well as some of the competitions in which they took part.

The traumatic pathology has been evaluated by registering the frequency, the gravity and the localization of the traumatic injuries according to an evaluation scale adapted from B. Montillet (1996). We have followed the distribution pattern of the traumatisms according to the location (arm, leg and body axis) and their type (fractures, sprains, muscular injuries) in the studied experimental group.

In the end, all the data have been sistematized in order to compare them and to see a general evolution of the traumatic pathology caused by the three kinds of performance sports. In order to capture the statistical significance of the differences which were noticed, the resulted statistical raw data processing was done.

**Results of the research and their interpretation**

During the follow-up period of the athletes an amount of 426 traumatic injuries were registered, on average 4,89 injuries/ athlete. However, not all the athletes suffered accidents followed by a temporary retirement from the sports activity, but only 106 athletes of 142, meaning 74,67% of them. Most of the accidents were light, to which the recovery was done relatively rapidly. 5% of the athletes with traumatic lesions were temporarily hospitalized, in order to be given a specialized medical care and to be subjected to the necessary investigations for the correct diagnosis and 53% of the athletes with traumatic injuries needed an interruption of the sports activity for minimum one week.

The arms represent together 18% of the surface of the body, the legs 36% and the body axis 54% [**19**]. Theoretically, each vulnerable agent can produce an injury to a certain segment according to the area of its surface at the level of the whole body surface (H_1_ hypothesis).

**Fig. 1:**
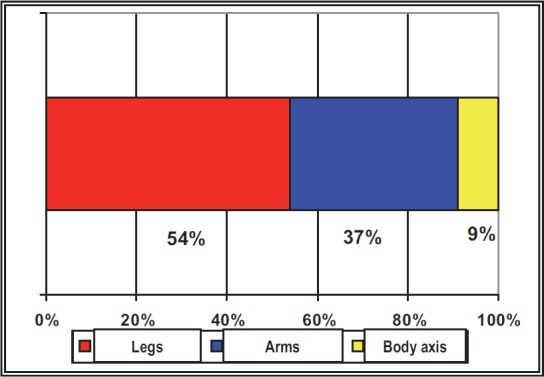
The comparative distribution of the traumatic injuries according to the localization

It was noticed that the serious traumatisms, such as the fractures, have been registered in a low percentage, the sprains, dislocations and muscular injuries being preponderant. The knee was the most affected articulation, followed by the articulations of the fingers from the hand, ankle and hip.

**Fig. 2:**
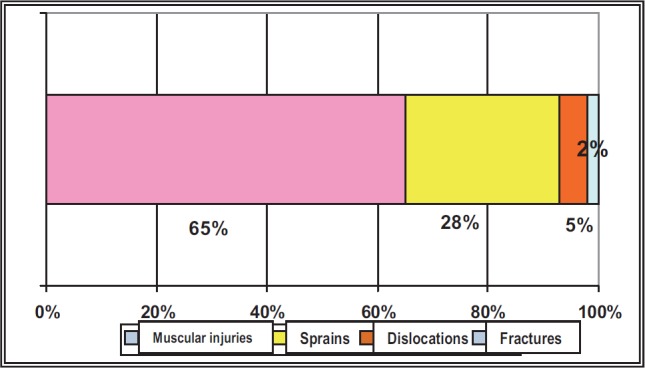
The comparative distribution of the traumatic injuries according to their types

The articular knee injuries have been the most serious (from the point of view of the severity). From the point of view of the production mechanism, most of the accidents have occurred in the context of counterattacks (injuries that occurred in offensive stages, approximately 33% of them).

**Fig. 3:**
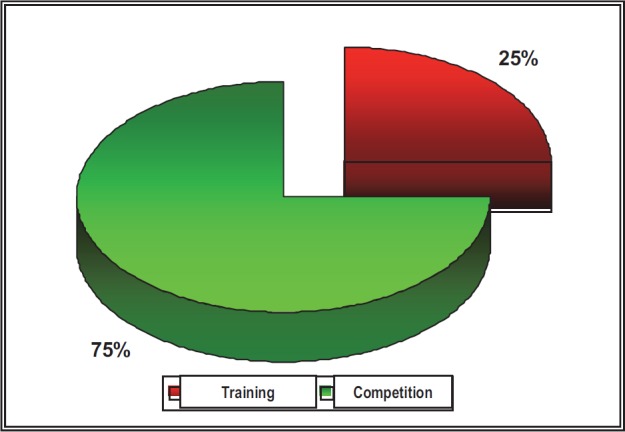
The comparative distribution of the traumatic injuries according to the moment they appeared (training/ competition)

Another aspect, which was followed in the study, was the establishment of the risk of recurrence of a traumatic injury, in other words, establishing the possibility of considering a muscular accident a potential risk factor for another accident in the same area (location).

**Fig. 4:**
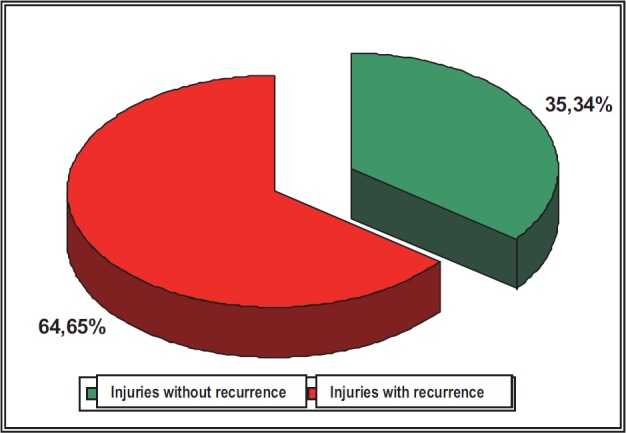
The comparative distribution of the traumatic injuries according to the presence or absence of the recurrence

## Conclusions

The incidence of the traumatisms of the locomotion apparatus in the three studied sports is higher during the competitions, and this situation is probably the consequence of approaching some specific competitive strategies.

A raise in the athletes’ degree of aggressiveness during the competitions, the most credible model, taking into consideration the level of engaging in the task, is conditioned by the anticipation of the reward and the ability of the athlete to evaluate the probability of task realization.

In the competition, due to the anxiety of the moment, mostly in the context of a structural anxiety, the loss or degradation of some technical elements takes place, which can lead to the production of the accident. Moreover, it is hard to state the moment the combativeness, which is wanted so much by the coaches, transforms in aggressiveness.

Although trainings have a higher weight than the competitions, and, during such training, difficult technical elements are learnt and exercised, they have a lower risk of accidents due to the lower level of affective participation.

However, it should be taken into consideration that the monitoring of the accidents is stricter during competitions compared to the trainings, and this takes its toll on the objectivity of the collected data. Moreover, the treatment is prompter and more correctly administered during the competitive sports events.

The traumatic injuries of the athletes appear mostly at the level of the most solicited segments, which are exposed to uncontrolled mechanical overloads, due to the synergic action of the muscles and some external factors (collision with an opponent, with the playing surface, etc.).

Usually, the mechanical overload to which the locomotor apparatus of the athlete is subjected during the physical activity is insufficient to cause traumatic injuries, but, in some cases, imbalances between the solicitation forces and the mechanical resistance of a segment intervene, thus accidents appear.
